# 

**Published:** 1893-03-04

**Authors:** 


					362 THE HOSPITAL? March 4, 1893.
OUR NEW OFFICES.
428, Strand, W.O., will henceforth be the Offioe of this Journal.
NOTICE TO CORRESPONDENTS.
All MS., Letters, Books for Review, and other matters intended foithe
Editor should he addressed Ths Editor, Tex Lodge, Pobohestbb
Square.London,W.
The Editor cannot undertake to return rejected M.S., even when
accompanied by stamped directed envelope,
Notice to Advertisers and Subscribers.
All Advertisements, Orders for Oopies of The Hospital, and Business
Oom annWvtions should be addressed to The Publisher, not to ths Editor,
Tii - -?..*pital, 428, Strand, W.O.
i?he Cost of Subscription, post free, is as follows:?Per week, 2$d. j
per quarter, 2s. 6d. ; per half-year, 5s. ; per annum, 8s. 6d,
Foreign??Per annum, 10s. 8d.; payable, in all cues, in advance.
Scraps and Gleanings.
A very BECiersful concert was held in the Prince of Wales Theatre,
Blackpool, in aid of the Blackpool Hoipital Fund.
It is now supposed that the actual digestion of carnivorous plants is
aided through the micro organism which are to be found in the sap of
plants.
A reply letter oard for international use has been invented by a
Frenchman, which would be of great service to correspondents if it
were brought into operation.
Cardinal Vaughan will be entrusted with an autograph letter of the
Pope to the Queen, which his HighnPss sends in recognition of what Her
Majesty has done for the Episcopal Jubilee.
'? The annual meeting of the working men's representatives in connection
with the Bury Dispensary Hospital, stated that last year the working
people raised ?1,223 16s. 55. towards that institution.
A new pier is being constructed at Southampton, and the authorities
have determined to spend ?9,000 on the pavilion aljne; how 1 jrg it will
take the 2d. toll to cover this expense it remains to be seen.
During the ensuing year the Belfast Lying-in Hospital enters on the
100th year of its existence, and it is proposed to hold a special meeting
in December to commemorate the foundation of the nstitution.
Physicians are now pronouncing that it is hiarhly injurious to
children's eyes to have lights burning in their bed-rooms; instaad of
allowing of perfect rest to their optic nerves, this keeps them in
motion.
The Committee of the Perth Royal Infirmary state that during the
past jear the district nurses have made 4 973 visits to patients,
ministering to their wants, and seeing that the instructions* ,of the
medical men were carried out.
The annual report of the Southampton Dispensary states that that
institution is, financially, in a good condition, and that it has benefitted
greatly through'the munificent donation of Mr. Barlow, and through the
bequest of the late Miss DumareBq.
The Committee of the B'ackkeath and Charlton Cottage Hospital
recorded the faot at their last annual meeting that Mr. Ashton'a
offering of ?100 had cleared the hospital of all debt, and the new year
was started with a clear balance-sheet.
The fund established in 1890 by Sir Smith Child for the benefit of fie
Staffordshire General Infirmary is now exhausted. The fever wards of
the establishment are closed for want of funds, and such money as
should go to capital is being used as income.
A large meeting of working men connected with the Working Men's
Hospital Committee was recently held at the Grimsby Hospital. The
gathering was held to mark the reopening of the Yarborough wing?
these new wards being inspected after all had partaken of tea.
The Datch have an excellent custom of calling attention to any hou^e
which has a case of infection in it, by tying a piece of white rag round
the bell handle. In the United States, a red card is often applied in the
case of scarlet fever, and in the case of small-pox, a yellow flag.
It is suggested that the ?1,000 which has been collected by the Con-
servative Benefit Society in order to erect a statue to the memory of
Mr. Holloway, should, instead, be applied for the use of the new ward
at the Stroud Hospital, and that it shall be called the " Holloway
Ward.
At the fifty-seventh annual meeting of St. Mark's Hospital, held at
the Mansion House, it was stated that want of fundi prevented the
committee carrying on the work of enlarging and modernising con-
sidered necessary for the establishment, for which purpose a freehold
property adjoining the present building had been purohasel.
Tor Student of Medicine and Medical Appointments see pages VIII. and IX. overleaf.
Books Received.
P. Blakiston and Co., Philadelphia.
" The Hygiene of the Sick-room." Bv W. B. Oanfield, M.D. 1892.
" Handbook of Massage." By Emile Kleen, M.D, 1892.
Longman, Queen, and Oo.
" Quain's Anatomy." Vol. III. Parti. Spinal Oord and Brain.
H. K. Lewis.
"A Pocket Medical^Dictionary." By George M. Gould, M.D.
Periodicals.?" The Gentlewoman" Offioe : "The Young Gentle-
woman." The Religious Tract Society: "The Sunday at Home,"
"The Girl's Own Paper." "Boy's Own Paper." "The Leisure
Hour." University and Pennsylvania Press: "Annals of Surgery."
Maomillan and Oo.: " The Practitioner."
viii THE HOSPITAL. March^, 1893.
The Student of Medicine.
[All comnmnicationB toe this Supplement should be addressed to The Editob of Xh? Hospital, at 428, dtt-and. with tue words. "Stnaeof
Column." written in the left hand top oorner of the envelope.!
APPOINTMENTS.
E. Beaumont, L.R.C.P.Lond., M.R.C.S., appointed Assistant
Medical Officer pro tern, to the Lewisham Board of Guardians.
J. S. Bristowe, M.D., F.R.C.P.Lond., F.R.S., M.R.C.S.Eng.,
appointed Consulting Physician to the Victoria Hospital for
Children, Queen's Road, Chelsea. G. Browning, M.R.C.S., re-
appointed Medical Officer of Health for the Stocksbride Urban
Sanitary District of the Wortley Union. M. 1'. M. Collier, M.S.
Lond., P.R.C.S.Eng., appointed Surgeon to the National Hos-
Sital for Diseases of the Heart, &c., Soho Square. E. B. Collings,
I.R.C.S., L.R.C.P.Lond., appointed Medical Officer of the
Workhouse of the Barnsley Union. J. W. Cook, M.D.Aberd.,
M.R.C.S.Eng., reappointed Medical Officer of Health for the
Tendring Rural Sanitary District. J. Cooper, M.R.C.S.Eng.,
L.R.C.P.Lond., appointed House Surgeon to the Great Northern
Central Hospital. A. K. Crossfleld, L.R.C.P., L.R.C.S.Edin.,
reappointed Medical Officer for the Dittisham District of the
Totnes Union. F. C. Dodsworth, L.R.C.P.Lond., M.R.C.S.Eng.,
reappointed Medical Officer of Health to the Chiswick Local
Board. A. Griffith, M.B., C.M.Edin, appointed Medical Officer
of Health for the Burgh, Falkirk. G. B. Hillman, L.S.A., ap-
pointed Medical Officer of Health for Whitwood. C. V. Hitchins,
M.R.C.S., appointed Medical Officer of Health for Weston-
super-Mare. E. Hogben, B.A., M.D., M.R.C.P., appointed
Medical Officer for the Parish of Strathmigh. R. B. Hogg,
M.R.C.S., appointed Senior House Surgeon to the Duuedin
Hospital, New Zealand. G. J. Brown Hope, M.A.St.And.,
M.B., C.M.Edin., appointed Medical Officer of the Hutton
Bushen District of the Scarborough Union. H. M. Kemmis,
L.R.C.P.I.. M.R.C.S., appointed Medical Officer for the No. 1
Sanitary District of the Bridgwater Union. T. Lakeman,
L.R.C.P.Lond., M.R.C.S.Eng., reappointed Medical Officer for
the Ugborough District of the Totnes Union. J. H. H. Manley,
M.B., B.C.Camb., M.R.C.S., D.P.H.Camb., reappointed Medical
Officer of Health for the County Borough of West Bromwich.
G. K. Pitcairn, M.B., C.M., reappointed Medical Officer of
Health for Littleborough. E. N. Reichardt, M.B.Lond., ap-
pointed Medical Officer for the Ewell, Chessmgton, ana y"
dington Sanitary Districts of the Epsom Union. H. D. Reynoldi ?
L.R.C.P.Edin., M.R.C.S.Eng., reappointed Medical Officer
Health for Pembroke Dock. A. Roche, M.R.C.P., L.R.C-?,A'j
appointed Examiner in Medical Jurisprudence in the
University of Ireland. J. T. Rowland, M.D.St.And., M.R-p-P'J
reappointed Medical Officer of Health for Richmond UrD
Sanitaty District. J. Shives, M.D. Aberd., L.R.C.S.Engo 1
appointed Medical Officer of Health to the Urban Sanitw
Authority of Liversedge. K. R. Smith, M.D.Loud., M.R-y' '
Eng., reappointed Medical Officer for the Halwell District of
Totnes Union. A. Harrison Thomas, M.B., C.M.,
appointed Medical Officer of Health for Vancouver, Brit
Columbia. A. Thomson, M.B., D.P.H.Camb., appointed Meal .
Officer of Health for Stratford-on-Avon Urban and RuraK>a
tary Districts. Lilian Trewby, L.R.C.P., L.R.C.S.E??'*
L.F.P.S.Glasg., appointed Senior House Surgeon to the jL?
Dufferin's Hospital at Agra, India. H. Ubsden, M.R.C.S-^ &?'
L.S.A., reappointed Medical Officer for the Staverton and R^' ,
District of the Totnes Union. J. H. Yinrace, M.B'L? ,'t
M.R.C.P., appointed Physician to the National Hospital
Diseases of the Heart, &c., Soho Square. J. K. Watson, '
C.M.Edin., appointed Souse Surgeon to the Morpeth Disp?11? J
W. T. G. Woodforde, M.D.Lond., appointed Medical Offic ^
Health for the Berkshire Combined Sanitary Districts. .
Young, M.B., C.M..Edin., Medical Superintendent of Brigk^j,
and District Joint Hospital, appointed Medical Officer of
for Brigbouse, Yorks.
VACANCIES. gt
The following vacancies are annonnocd this week, the amonnt
salary in eaoh case being given after the name of the institution:
Resident Medical Officers. ^
Oity of London Hospital for Diseases of the Ohest, Victoria Pa^iu(?
House Ptiysioian. Board, residence, and allowanoe for JJr* j,ory
provided. Applieations to the Secretary, at the offlue, 21,-r1110
CJircus, E.G., by Maroh 9ih. f
March 4, 1893. THE HOSPITAL.
IX
THE STUDENT OF MEDICINE?(continued).
General Hospital, Birmingham. House Physician. Paliry ?70 per
annum, with residence, board, and washinpr. Applications to
Howard J. Collins, House Governor, by March 25th.
Cancer Hospital (Free), Fulham Road. 8.W. House Surgeon. Appoint-
ment for six months. Salary at the rate ot ?50 per annum, with
board and residence. Applications to the Secretary by March 18th.
Hnddersfield Infirmary. Junior House Surgeon. Salary ?40 per annum,
with board, lodging, and washing. Applications to Mr, Joseph Bate,
Secretary, by Maroh 4th.
London Temperance Hospital, Hampstead Road, N.W. House Surgeon.
Doubly Qualified. Appointment for six months. Salary at the
fate of 50 guineas per annum, with residenoa in the hospital, board
and washing. Applications to E. Wilson Taylor, Secretary, by
T March 3rd. . ?
OEdon Temperance Hospital, Hampstead Road, N.W. Junior House
Surgeon. Appointment for six months. Residence in the hospital,
board and washing provided, and an honorarium of 5 guineas at
satisfactory completion of term of appointment. Applications to
*? .J* Wilson Taylor, Secretary, by March 3rd. t . ,
"Othngham General Dispensary, Broad Street, Nottingham. A?sistant
Resident Surgeon; doubly qualified. Salary ?150 per annum, with
rooms and attendance. Applications to Charles H. Preston,
Di. Secretary, by March 6th. ? ,
lnford, Rutland, and General Infirmary. House Surgeon and
Secretary. Unmarried. Donbly qualified. Appointment for two
years. Salary ?100 per annum, with board, lodgifcg. and washing.
Applications to "The Chairman of the Special Commit*, eo, by
March 7tb. v
Non-Resident Medical Officers.
ristol Dispensary, Castle Green, Bristol. Vacancy in the Medical Staff,
double qnalificition. Applications to Edward Stock, Secretary, by
March 10th.
t? H?spital, S.E. Assistant Dental Surgeon. Applications to the
If,,. 0an'by March 4th. _ _ ?
Ho?pital, Queen's Square, Bloomsbary, VV.O. _ Honorary
J hyaician. Applications to the Honorary Secretary, Arthur Serena,
P?v?-,AusUn Fri.rs, E.C. . . .
a Dispensary, 59, Stanhope Street, Clare Market.
Applications to John Phillips, Secretary, 14 and 15, Portugal htreet,
a M'coln's Inn, by Maroh 6th. . , n j
?ri*an Frea Hospitil for Women and Children, Marylebone Road,
Physician to Out-Patient Department. Applications to
8t \i80rg.e Scudamore, Secietary. . . , ,
" ""7 s Hospital Medical School. Demonstrator in Chemistry and
j^ysics. Salary ?100 per annum. Applications to George P. i lela,
University of Glasgow. Four examiner,-hip* for degrees in Medio in?
Annual fee, ?30. Applications to Alau E. Olapperton, Secretary'
to the Court 91, West Regent Street, Glasgow, by March 11th.
Yorkshire College, Leeds. Demonstrator in Anatomy. Applications to
the Rsgistrar.
PASS LIST.
University or Iiondon.
INTERMEDIATE EXAMINATION IN MEDICINE.?January, 1893.
Entire Examination.?First Division.?G. J.Branson, B.A., Mason
College; A. W. R. Coohrane, St. Bartholomew's Hospital; R. Hopton,
Yorkshire Oollega. Second Division.?P. E. Adams, St. Bsrthol >mow's
Hospi alj J. A. Atkinson, St. Mary's Hospital; C. Banting. University
College; P. C. Barford, St. Bartholomew's Hospital; F. M. Burnett,
St. Bartholomew's Hospital; J. E. G. Calverley, St. Bartholomew's
Hospital; F. Ohown, St. Mary's Ho p tal; J. Coken. London Hospital;
E. Coleman, Guy's Hospital; F. J, Contts, University College; A. P.
Cammings, Yorkshire College; C. S. DeSegundo, St. Bartholomew's
Hospital; W. E. N. Dunn.St. Bartholomew's Hospital; a. H. Gerrard,
University College; T. Hampton,St. Bartholomew's Hospital; W. E.L.
Horner, Un versity College; R. Hughes, St. Thomas's Hospital; T. H.
Hunt.Owens and Yo kshire Colleges ; F. H. Jacob, King's College; A.
Marriott. University College; W. P. Nicol, Mason College; F. S. Penny,
King's Coll)ge; G. B. Price, Bristol Medical School and Clifton
Laboratory ; M. H. Raper, Middlesex Hospital; H. M. Bigby, London
Hospital; S. G. Tippett, Westminster Hospital; R. H. Townend, London
Hospital; T. H. Wells, Middlesex Bospital and Birkbeck Institute j
F. K. Wi son, Wentminster Hospital.
Excluding Physiology.?First Division.?E. B. Bos^ock, Mason
College; A. W. Jenkins, University College. Second Division.?W.
Allpoit, Mason College; C. H. Caldicott, Mas?n Oolltge; M. Dick,
University College ; A D. Due it, St. Bartholomew's Hospital; W. H.
Holliday. Yorkshire C ?kge; D. Horwitcli, Mason College; A. E.
Hatton, Yorkshire College ; R. M. Johnston, University College; E. T.
Jones, University College; 0. H. Mott, London Hospital; M. Wilts,
University College.
Physiology Only .?First Division.?A. Hunnard, University College.
Second Division.?J.N. Brown, University College; J. S. Choter, St.
Bartholomew's Hospital; G. W. Gostling, University College ; M. L. G.
Hallwrieht, Mason College ; G. R. Harcourtt, Ht. Thomas's Hospital;
F. D. Harris, St. Mary's Hospital; S. P. James, St. Mary's Hospital;
Amelia M. Le Pelley, London School of Medicine for Women ; J. Mace,
Yorkshire College; W. M. Price, Guy's Hospital; A. E. Reynolds,
University College; S. E. Shoppee, University College; A. Tait, York-
shife College; R. H. Tompsett. St. Thomas's Hospital; A. Young,
Sheffield Medical School and University College.

				

